# Unveiling the Interfacial Dynamics of Crosslinked Biodegradable Polycaprolactone Solid Polymer Electrolytes and Li Metal During Lithium Plating and Stripping *via Operando* ATR‐FTIR Spectroscopy

**DOI:** 10.1002/advs.77788

**Published:** 2026-09-16

**Authors:** Jian‐Fen Wang, Matthias Weiling, Felix Pfeiffer, Marian C. Stan, Martin Winter, Masoud Baghernejad

**Affiliations:** ^1^ Helmholtz Institute Münster: Ionics in Energy Storage (IMD‐4 / HI MS) Forschungszentrum Jülich GmbH Corrensstrasse 48 Münster Germany; ^2^ MEET Battery Research Center University of Münster Corrensstraße 46 Münster Germany

**Keywords:** interphase characterization, lithium metal anode, *operando* ATR‐FTIR spectroscopy, polycaprolactone polymer electrolytes, solid‐state batteries, zero‐excess batteries

## Abstract

Polycaprolactones (PCL) have gained attention as a biodegradable and environmentally sustainable solid polymer electrolyte (SPE) for solid‐state battery applications. While poly(ethylene oxide) (PEO) systems are widely studied, the interfacial mechanisms of PCL‐based SPEs in contact with Li metal remain insufficiently explored. In this work, *operando* attenuated total reflection Fourier‐transform infrared (ATR‐FTIR) spectroscopy was employed to monitor the chemical environment at the interface of Li metal and crosslinked PCL‐based SPEs (cl‐PCL_5_LiTFSI) during Li plating/stripping, enabling both spectral assignment and electrochemical comparison between the crosslinked and non‐crosslinked systems. During galvanostatic cycling experiments, cells with cl‐PCL_5_LiTFSI SPEs exhibited increased cycling stability compared to cells containing PCL_5_LiTFSI SPEs, which can be attributed to the enhanced mechanical strength of the polymer network. Overall, the results show the development of localized ordered PCL domains and the redistribution of TFSI^−^ anion during Li plating/stripping, revealing the possible degradation and dynamic changes occurring at the SPE|Li metal interface. The phenomenon of interfacial evolution of the SPEs was visualized through converting specific characteristic bands of PCL and LiTFSI from *operando* spectra into penetration depth‐dependent band ratio maps.

## Graphical abstract

This study presents a comprehensive interfacial characterization of the biodegradable PCL solid‐polymer electrolytes (SPEs) with Li metal, utilizing an advanced *operando* ATR‐FTIR spectroscopy. The investigation elucidates the interphase properties during Li plating/stripping in a zero‐excess cell configuration. The results were further converted into band intensity‐ratio map in order to visualize the correlation between penetration depth and Li plating/stripping status and unveiled the dynamic evolution of PCL crystallinity change and TFSI anion redistribution.

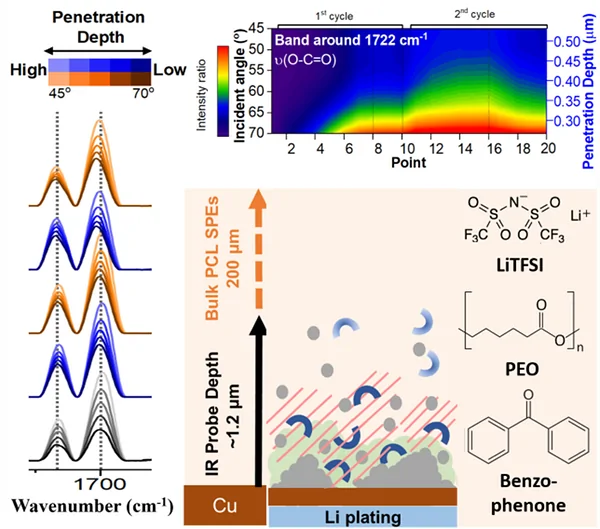

## 1 Introduction

Lithium‐metal batteries (LMBs) are promising candidates for high energy density energy storage systems due to the use of Li metal, providing exceptionally high theoretical capacity (3860 mAh g^−1^) and the lowest standard reduction potential (−3.04 V vs. standard hydrogen electrode, SHE) [1, 2, 3]. Among LMBs, zero‐excess configurations present a boost in energy density, as the layout after cell assembly consists solely of a bare current collector without any active material, thereby reducing both mass and volume for the batteries [4, 5, 6]. Solid‐state electrolytes (SSEs) enable the use of LMBs, emerging as a prospective alternative to liquid electrolytes, which address the safety concerns associated with the flammability and chemical stability of the latter [7, 8, 9, 10]. Especially, solid polymer electrolytes (SPEs) are valued for their higher mechanical flexibility, ease of preparation, and improved interfacial contact with the electrodes compared to inorganic solid electrolytes [11, 12].

Among the SPEs, poly(ethylene oxide) (PEO) SPEs have been extensively studied and developed into flexible membranes due to their excellent coordination with Li conductive salts and structural simplicity for side‐chain modification [13]. Building on these advantages, polycaprolactone (PCL) has attracted increasing attention due to its low glass transition temperature (*T*_g_ = −60°C) and the interaction between the carbonyl groups of PCL and a Li ion is much weaker compared to the ether‐Li ion interaction in PEO. This results in improved Li ion mobility and consequently the Li ion transference number (*t^+^*) from 0.2 to 0.5 [14, 15, 16]. Particularly, PCL SPEs stand out due to their most relevant feature of biodegradability, which makes PCL a promising material for future environmentally sustainable battery technologies [14, 17]. C.P. Fonseca et al. were the first to report a system based on PCL and LiClO_4_, which underwent complete biodegradation after 100 days [14]. This promising feature of biodegradability makes PCL attractive for application in next‐generation sustainable batteries [18]. However, the ionic conductivity of PCL with 27 wt.% of lithium bis(trifluoromethanesulfonyl)imide (LiTFSI) at 30°C has been reported to be in the order of 10^−5^ to 10^−7^ S cm^−1^ [19, 20, 21]. This poor ionic conductivity at 30°C arises primarily from PCL's semi‐crystalline structure, which has low segmental mobility under ambient conditions that further restricts Li‐ion transport. Furthermore, additional limitations have been identified for PCL SPEs, including insufficient mechanical strength and thermal stability above their melting point of 60°C, compromising the performance and safety of the battery [22].

Various modification strategies have been developed to overcome these thermal and mechanical limitations. The most reported approaches include crosslinking, copolymerization, and incorporation of inorganic fillers to simultaneously suppress crystallinity and improve the mechanical properties of PCL polymers [20, 23, 24, 25]. Crosslinking offers several distinct advantages for polymer modifications [19, 26, 27, 28, 29]. Among them, the use of ultraviolet (UV) crosslinking initiators is a straightforward and scalable method to construct a multidimensional polymer network [28, 30, 31, 32]. It was shown that benzophenone (BP), used as a UV‐photoinitiator for PEO crosslinking, remained in the matrix after crosslinking and formed a reversible complex with Li metal in our previous study [33]. While exposure of BP to excess Li metal led to unstable complexes and degradation, the crosslinked PEO SPEs still showed improved chemical stability over non‐crosslinked systems [34]. Thus, this photo‐initiated crosslinking method is advantageous, although it should be applied with a minimum effective dosage and voltage window in which BP remains stable in the SPEs.

The investigations of the PCL‐based SPEs|Li metal interface have been conducted mainly through theoretical modeling, and a few were supported by experimental characterization. Ebadi et al. employed density functional theory (DFT) calculations to reveal strong adhesion and charge redistribution at the PCL|Li interface, suggesting a tendency of PCL degradation and formation of a solid electrolyte interphase (SEI) [35]. Complementary, ab initio molecular dynamics (AIMD) simulations carried out by Wu et al. elucidated the decomposition mechanism of the PCL+LiTFSI electrolyte on Li metal surfaces, identifying key SEI species such as LiF from LiTFSI degradation and Li_2_O/carbonaceous fragments from PCL degradation [36]. Andersson et al. revealed through DFT calculations that the C─O bond in PCL is relatively prone to cleavage upon contact with Li metal [37]. Correspondingly, ex situ x‐ray photoelectron spectroscopy (XPS) analysis detected the formation of Li_2_CO_3_ and Li carboxylate species, confirming interfacial decomposition of PCL in the presence of Li metal. Besides, ex‐situ spectroscopic analyses have also contributed valuable perspectives. Woo et al. used Fourier‐transform infrared (FTIR) spectroscopy to study ion association in PCL‐based proton conductors, establishing a correlation between the ammonium thiocyanate (NH_4_SCN) salt concentration and ionic conductivity [38]. In a related study, Phillipson et al. characterized the crystallization kinetics of neat PCL indicated by shifts of the carbonyl bands when employing FTIR spectroscopy, offering a detailed view of polymer phase transitions without incorporating lithium salts or electrode interfaces [39]. Together, these studies form a strong foundation for understanding PCL behavior across multiple contexts. However, the experimental validation under real battery operating conditions is still insufficient.

Herein, we provide complementary *operando* spectroscopic evidence of the interfacial evolution in PCL‐based SPEs during the Li metal plating/stripping process. To achieve this, a comprehensive analysis including electrochemical experiments and *operando* ATR‐FTIR spectroscopy measurements of PCL‐based SPEs was conducted. *Operando* ATR‐FTIR spectroscopy measurements are further employed to monitor interfacial changes in real‐time using the spectro‐electrochemical cell with a reservoir‐free configuration setup, to acquire valuable insights into the PCL SPEs|Li metal interface mechanism under real working conditions [33, 40]. Additionally, a penetration depth‐dependent band intensity ratio map analysis is presented to elucidate the spatial distribution of chemical species and reactions across the interface with Li metal at an elevated temperature of 60°C.

## 2 Results and Discussion

### 2.1 Spectra Analysis of Bulk PCL‐Based SPEs

Accurate spectral assignment is critical for interpreting the band origin of the SPEs, as the addition of Li salts and the crosslinking in the polymer can cause changes in the chemical environment of the materials, resulting in a shift of the corresponding bands. Although previous studies have reported vibrational assignments of pure PCL, the use of PCL in an SPE with Li salt could lead to misinterpretation of the spectrum. Therefore, establishing a composition‐dependent spectral dataset is essential for reliable analysis.

To establish a database for spectral interpretation and provide a reference for subsequent *operando* analysis, bulk infrared spectra of the materials of SPEs were first examined at room temperature. Bulk materials, SPEs with different PCL:LiTFSI ratios (Li:C = O ratio of 1:10 and 1:5), both with and without crosslinking, were compared to identify the origins of the characteristic bands. The corresponding spectra and band assignments are presented in Figure S1 and summarized in Table S1. Additionally, the ionic conductivity of these SPEs (PCL_10_LiTFSI, cl‐PCL_10_LiTFSI, PCL_5_LiTFSI, and cl‐PCL_5_LiTFSI) across a temperature range of 30‐70°C are summarized in Table S2 and further discussed in the Supporting Information.

As shown in Figure S1a, upon the addition of LiTFSI to PCL polymer, several characteristic IR bands shift or overlap, reflecting changes in molecular interactions and coordination environments. By systematically comparing spectra of the SPEs from low (PCL_10_LiTFSI) to high LiTFSI concentrations (PCL_5_LiTFSI), the underlying causes and origins of these spectral changes can be clarified. In Figure S1b,c, a considerable portion of the bands are situated in these areas, leading to band overlap and mutual interference. Therefore, several notable band shifts and the overlapping of the bands were highlighted. For instance, the characteristic bands corresponding to bulk LiTFSI were at around 810 cm^−1^, 773 cm^−1^, and 745 cm^−1^, but these shift to 790 cm^−1^, 762 cm^−1^, and 740 cm^−1^, respectively, when LiTFSI is embedded in the SPEs.

**FIGURE 1:**
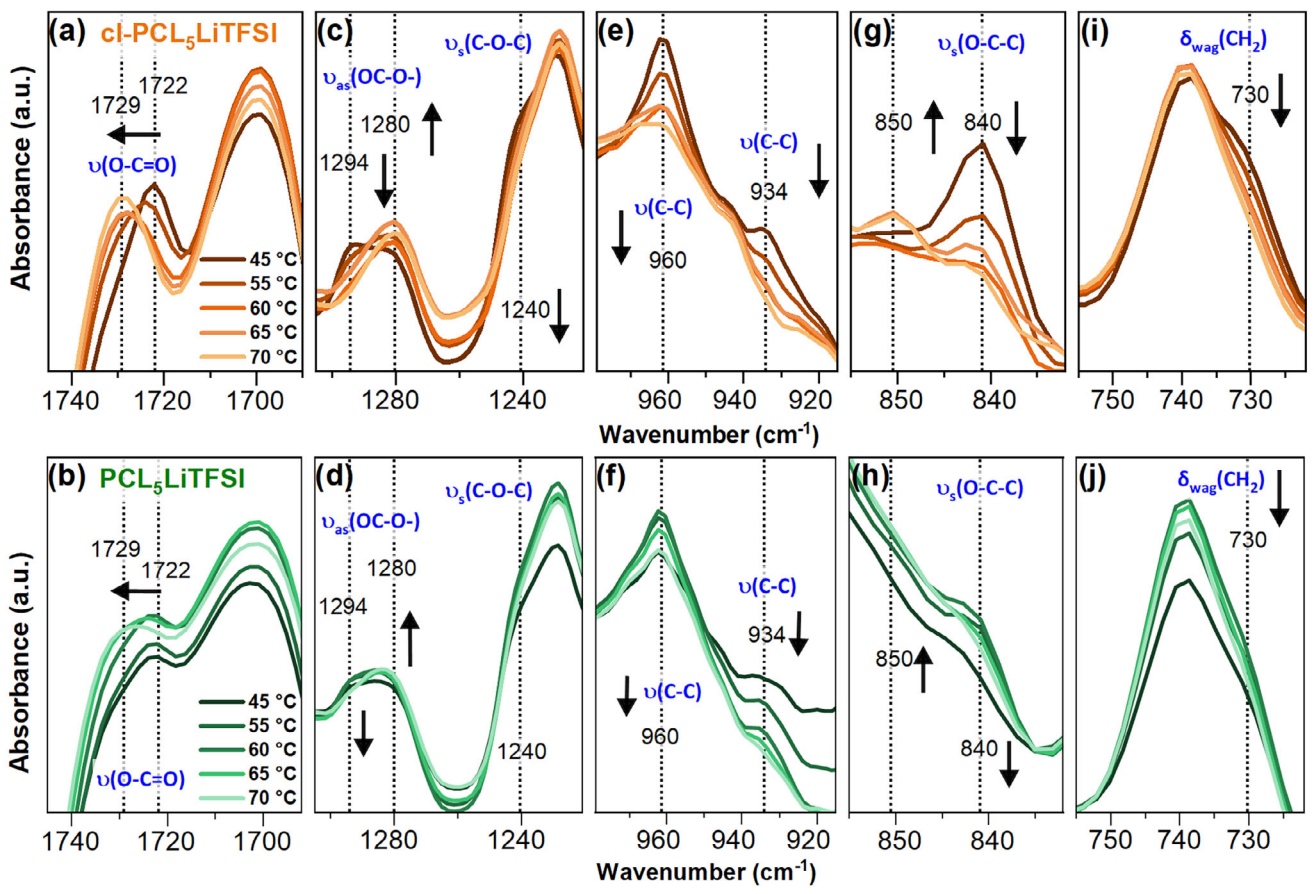
Temperature‐dependent IR spectra of the cl‐PCL_5_LiTFSI and PCL_5_LiTFSI SPEs measured at temperatures from 45°C to 70°C (color gradient from dark to light). The selected spectral regions are primarily associated with the vibrational modes of PCL polymer and reflect changes in crystallinity changes: (a,b) selected spectra region of 1740−1700 cm^−1^ (c,d) selected spectra region of 1310−1235 cm^−1^ (e,f) selected spectra region of 980–915 cm^−1^ (g,h) selected spectra region of 860–835 cm^−1^ (i,j) selected spectra region of 750−720 cm^−1^. The arrows represent the tendency of the band intensity to increase, decrease, or shift.

Previously, we observed that crystallinity transitions of the PEO polymer during Li plating at the PEO SPEs|Li metal interface can be observed by distinct changes in the IR spectral features [33]. By Differential Scanning Calorimetry (DSC) investigations comparing pure PCL, PCL_5_LiTFSI, and cl‐PCL_5_LiTFSI SPEs (see Figure S2) the melting temperatures were analyzed, revealing that the SPEs retain partial crystallinity after crosslinking. Additionally, the temperature‐dependent experiments were carried out in our in‐house‐designed, temperature‐controllable spectro‐electrochemical cell, with the samples measured directly on a ZnSe ATR crystal. Identifying IR bands of PCL SPE influenced by crystalline phase changes is useful for interpreting structural evolution during electrochemical processes. Although the infrared spectral assignments regarding the crystalline and amorphous phase of pure PCL polymers were thoroughly established by K. Phillipson et al., those of its electrolyte form (PCL SPEs) remain completely uncovered, as the absorption bands shift upon the addition of lithium salt due to ion‐polymer coordination. The temperature‐dependent experiment involved heating the cl‐PCL_5_LiTFSI and PCL_5_LiTFSI SPEs from 45°C to 75°C, and the spectra were collected after thermal equilibrium at each temperature. Figure 1 shows the selected spectral regions where changes in band position or intensity were observed at elevated temperatures. The full spectra are provided in Figure S3.

At 45°C, the IR spectral features are identical to those measured at room temperature (as shown in Figure S1), suggesting that no phase transition has yet occurred and the crystalline phase of PCL remains predominant in the polymer electrolyte. As the temperature increases, gradual changes are observed in the selected spectral bands shown in Figure 1. The bands around 1722, 1294, 1240, 960, 840, and 730 cm^−1^ decrease in intensity with increasing temperature (Figure 1a–j) indicating a decrease in PCL crystallinity. In particular, the bands around 1729, 1280, and 850 cm^−1^ increase in intensity, which is assigned to a growth in the amount of amorphous PCL phase. The spectra of the non‐crosslinked PCL_5_LiTFSI SPEs at 45°C exhibit overall lower absorbance intensity. This effect likely originates from poor contact with the ATR crystal, since the PCL_5_LiTFSI SPEs membrane remains stiff at this temperature, leaving small gaps that alter effective evanescent wave penetration, resulting in lower intensities. However, the overall intensity increases as the SPEs soften and have better contact when the temperature rises, following the same general trend as the crosslinked cl‐PCL_5_LiTFSI SPEs.

Despite following similar trends, the performed FTIR characterizations indicate that the PCL_5_LiTFSI SPEs require a higher temperature to reach the same amorphous‐phase band features compared to the cl‐PCL_5_LiTFSI SPEs. Furthermore, it should be considered that the aforementioned wavenumbers were determined based on the intensity maxima positions of the bands, which may vary due to the overlap of the closely positioned bands. For example, the band shift observed between 1722 and 1729 cm^−1^ could result from changes in the relative proportion of crystalline and amorphous phases of PCL.

### 2.2 Electrochemical Performance of the PCL Electrolyte in the Reservoir‐Free Configuration Coin Cells

To investigate the (electro‐)chemical stability of PCL toward plated Li metal, coin cells containing Li metal and perforated Cu foil electrodes (Li|SPE|Cu) were prepared, including PCL_5_LiTFSI or cl‐PCL_5_LiTFSI SPE membranes. The non‐crosslinked PCL_5_LiTFSI electrolyte with its known intrinsic mechanical and chemical failure behavior was employed as a reference system to demonstrate the beneficial effect of cross‐linking PCL_5_LiTFSI.

**FIGURE 2:**
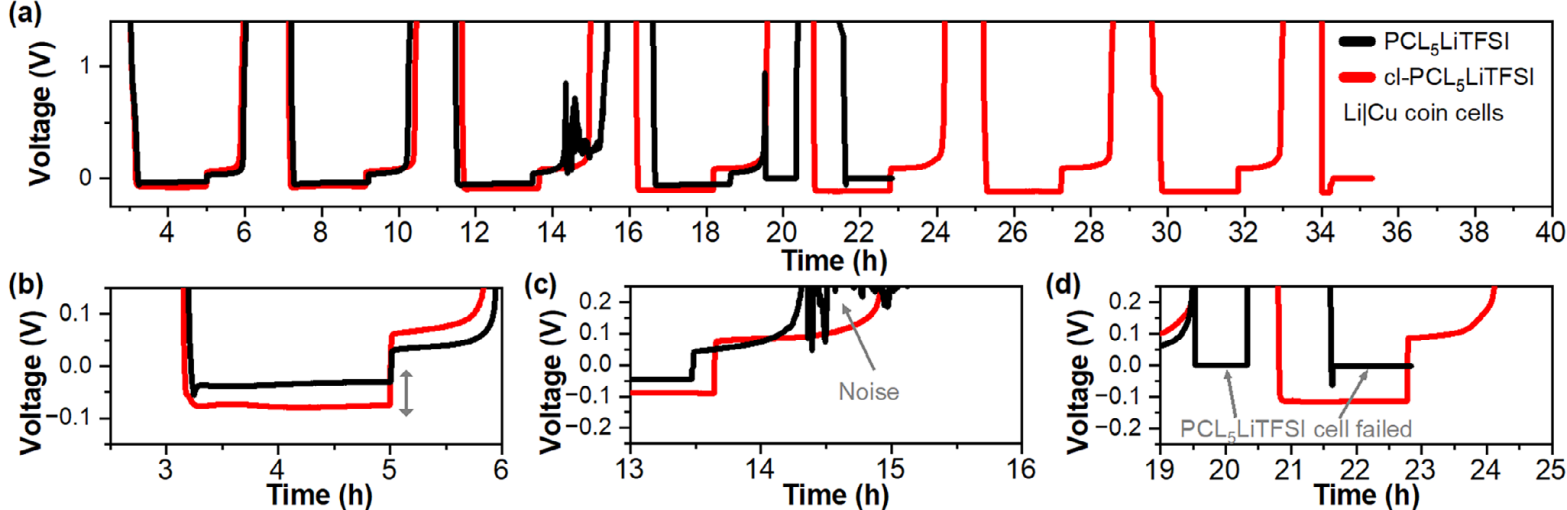
Li plating/stripping cycles (current density of 0.05 mA cm^2^) in the reservoir‐free configuration Cu||Li coin cells at 60°C. (a) Comparison of cycling performance of cells with cl‐PCL_5_LiTFSI and PCL_5_LiTFSI SPEs. (b) Overvoltage difference during the first Li plating/stripping cycle for cells with cl‐PCL_5_LiTFSI and PCL_5_LiTFSI SPEs. (c) Noise points observed during the third Li stripping cycle in the cell with PCL_5_LiTFSI SPEs. (d) Cell with PCL_5_LiTFSI SPE failed during the fourth Li plating half‐cycle. All cell variations were repeated four times and the cells with the longest lifetimes are shown here.

The results presented in Figure 2 enable a direct comparison between cells containing cl‐PCL_5_LiTFSI and PCL_5_LiTFSI SPEs during Li plating/stripping cycles. In Figure 2a, the cell containing a cl‐PCL_5_LiTFSI SPE exhibits a longer lifetime during Li plating/stripping cycles. In addition, the cell with cl‐PCL_5_LiTFSI SPE exhibits an overall higher overvoltage (−75 mV) compared to the cell with the PCL_5_LiTFSI SPE (−45 mV, Figure 2a,b). The increased overvoltage can be attributed to the crosslinked network formation, restricting segmental mobility and thereby hindering effective Li ion transport. As shown in Figure 2c,d, the cell containing PCL_5_LiTFSI SPE exhibits distinct voltage noise at the third Li stripping cycle and subsequently fails at the fourth Li stripping cycle. The shorter lifetime of cells containing the PCL_5_LiTFSI SPE is primarily attributed to its poor mechanical strength, allowing the formation of Li dendrites penetrating the SPE, which ultimately triggers short circuits.

As shown in the image of the opened coin cells in Figure S4, the PCL_5_LiTFSI SPE membrane exhibits insufficient mechanical strength and undergoes pronounced thickness redistribution and deformation under stack pressure. In contrast, the cl‐PCL_5_LiTFSI SPE forms a network that better withstands local stack pressure and effectively prevents dendrite penetration.

**FIGURE 3:**
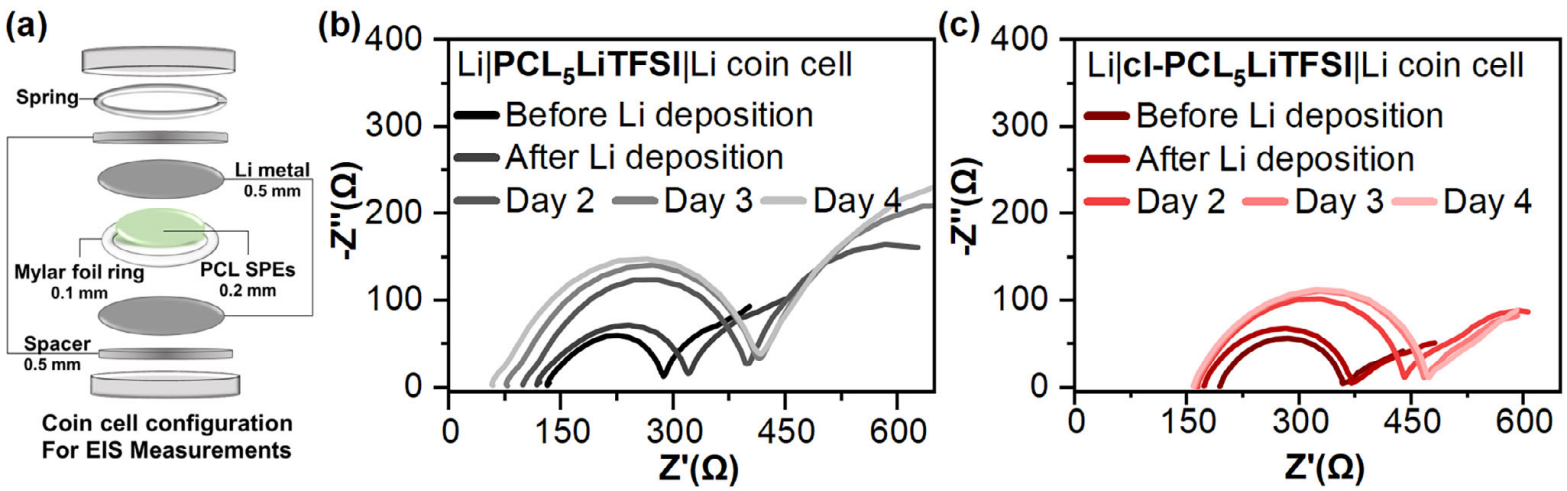
(a) Coin cell configuration employed for EIS measurements, which includes an additional Mylar foil ring with a hole the same size as the SPE membrane. This ring acts as a spacer to prevent short circuits arising from direct contact of two Li electrodes and insufficient mechanical strength of the non‐crosslinked SPEs. (b) Nyquist plots of the Li||Li cells containing PCL_5_LiTFSI SPE. (c) Nyquist plot of the Li||Li cells containing cl‐PCL_5_LiTFSI SPE. Measurements were taken before and after 2 h of Li plating at a current density of 0.05 mA cm^−^^2^, and remeasured after 24, 48, and 72 to monitor the evolution of the impedance over time.

Electrochemical impedance spectroscopy (EIS) was conducted on Li||Li coin cells in order to measure the intrinsic SPE|Li interfacial impedance under defined and reproducible conditions. To mitigate the poor mechanical strength issue of PCL_5_LiTFSI SPEs and focusing on the contacting interface of the SPE|Li metal, an additional Mylar foil ring as a mechanical spacer was introduced to the cells to maintain a minimum distance between the Li metal electrodes, as shown schematically in Figure 3a.

The first and second EIS measurements were carried out before and after Li plating at 0.05 mA cm^2^ for 2 h at 60°C to provide fresh Li metal at the interface. Afterward, EIS measurements were carried out after resting for 24, 48, and 72 h, in order to investigate the evolution of the interfacial impedance between the plated Li metal and the two types of SPEs. The Nyquist plots shown in Figure 3b,c, were fitted using the equivalent electric circuit presented in Table S3, where the impedance values from the cells containing PCL_5_LiTFSI and cl‐PCL_5_LiTFSI SPEs are also reported. After 2 h of Li plating, both cells exhibited higher resistances, which may be attributed to the SPEs’ degradation occurring during the Li plating. After 24 h of rest, a pronounced increase in interfacial resistance was observed in both cells. Moreover, impedance growth became more moderate yet constant after 48 and 72 h, indicating that the SPEs still undergo degradation. When comparing the fitted values (Table S3), the cell containing cl‐PCL_5_LiTFSI SPEs exhibits a larger initial impedance, which is consistent with the higher overvoltage observed in Figure 2a,b. In contrast, the cell containing PCL_5_LiTFSI SPE shows a more pronounced increase in impedance after Li plating and after resting for various periods of time, indicating enhanced SPE degradation and possibly SEI growth. Notably, the R1 value (bulk resistance) from the cells containing the PCL_5_LiTFSI SPE decreased gradually over time, which might be due to the decrease in the thickness of the SPE membrane under stack pressure, as depicted in Figure S4a,b, where PCL_5_LiTFSI SPE was extruded by internal pressure, leaving an uneven thin layer between the Li electrodes.

The EIS results indicate that upon exposure to electroplated Li metal, the non‐crosslinked PCL_5_LiTFSI SPE underwent the most pronounced degradation after 24 h, leading to the formation of a resistive interphase. Although the rate of degradation slowed down after 48 and 72 h, impedance steadily increased, indicating an unstable SEI formation. In contrast, the cl‐PCL_5_LiTFSI SPE also exhibited its highest degradation at 24 h, but the extent of degradation decreased substantially at 48 and 72 h, resulting in marginal changes in resistance. This finding suggests that crosslinking effectively mitigates the intrinsic tendency of PCL to undergo extensive degradation in contact with Li metal, which is consistent with the observed cycle‐life results.

### 2.3 PCL SPE Interphase Evolution Investigation *via Operando* ATR‐FTIR Spectroscopy

The evolution of the interphase formed by the possible reaction of PCL SPE with plated Li metal was investigated by means of *operando* ATR‐FTIR measurement using the inhouse designed spectro‐electrochemical cell setup [30].

**FIGURE 4:**
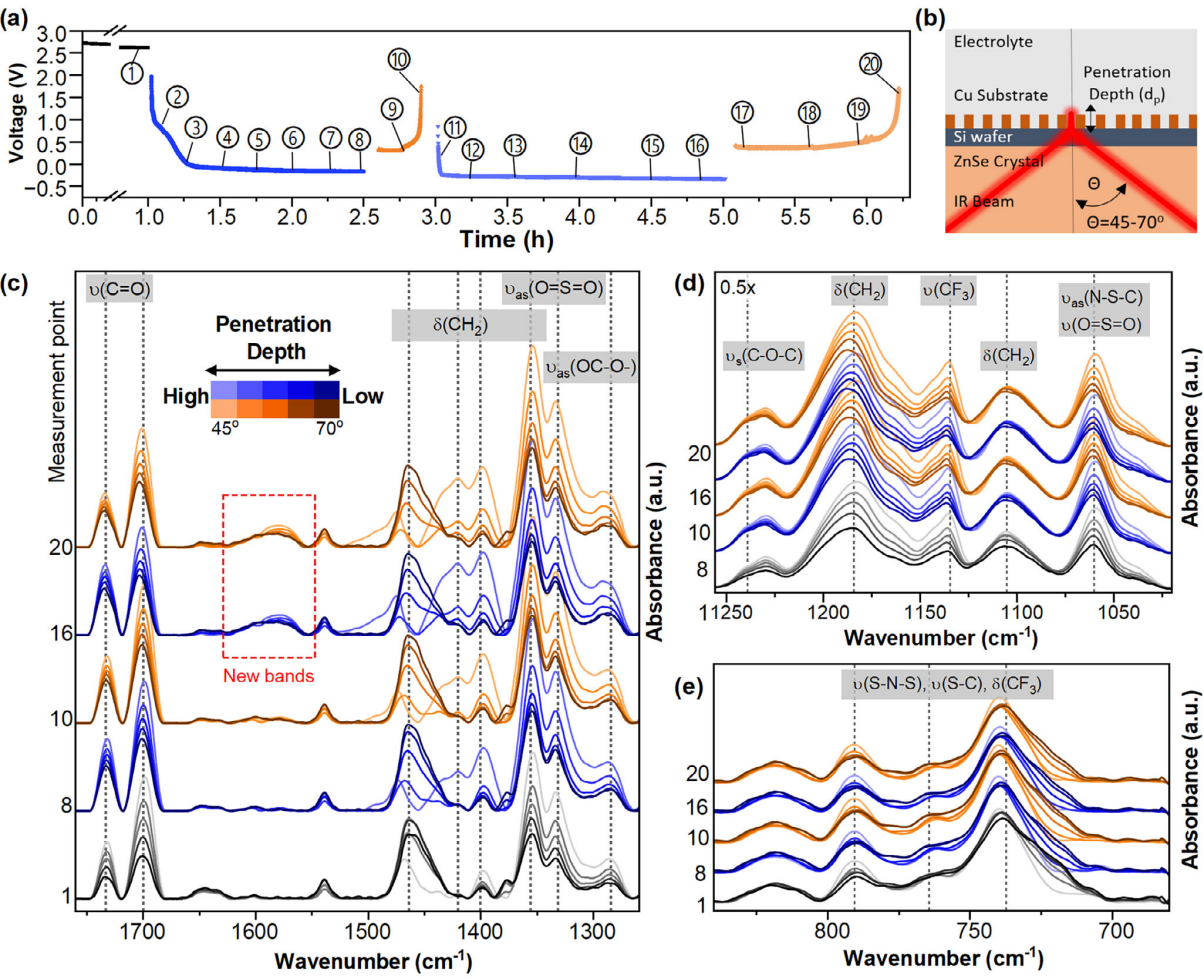
(a) Voltage vs. time profile of Li plating/stripping cycles at a current density of 0.05 mA cm^−2^ in the spectro‐electrochemical cell containing Li|cl‐PCL_5_LiTFSI SPEs|Cu at 60 °C. IR spectra were recorded during the electrochemical process (points 1‐20). (b) At each corresponding state, IR measurements were performed with incident angles from 45° to 70°, enabling depth profiling. (c–e) ATR‐FTIR spectra of points 1, 8, 10, 16, and 20, representing each state of the electrochemical process. The shift from dark to light color represents the increase in penetration depth. (c) Spectra range of 1760–1260 cm^−1^ (d) Spectral range of 1255–1020 cm^−1^ (e) spectral range of 840–680 cm^−1^.

The results, together with the stages at which the IR measurements were recorded (Points 1‐20), are presented in Figure 4a. During Li plating/stripping cycles, ATR‐FTIR spectra were recorded simultaneously at each stage of the electrochemical process. The schematic overview in Figure 4b shows the cell configuration and IR beam path in the ZnSe crystal at variable incident angles (45°‐70°), resulting in a change in penetration depth into the SPEs.

Depth‐dependent interphase information was obtained, from higher penetration depth at lower angles to shallower penetration depth at higher angles. The conversion between IR incident angle and penetration depth follows the Harrick equation of evanescent field and penetration depth, as shown in Equation (1) in the Experimental Section [41, 42, 43]. Notably, a lower wavenumber would result in higher penetration depth, thus providing information closer to the bulk SPE, whereas a higher wavenumber at the same incident angle results in lower penetration depth and provides details closer to the SPE|Li metal interface. Detailed theoretical background and the explicit formulas used for the numerical conversions are also provided in the Experimental Section. Additionally, the *operando* investigation of the non‐crosslinked SPE (PCL_5_LiTFSI) is presented for comparison (Figures S5–S7), along with the discussion of the spectra in the SI.

As shown in Figure 4a, the first cycle resulted in a lower Coulombic efficiency (CE) of 25% compared to the coin cell results given in Figure 2, presenting a CE of 51% in the first cycle. This indicates that the majority of the deposited Li was consumed in parasitic side reactions. This loss may originate from interfacial degradation of the SPEs in contact with Li metal and the reduction of native Cu oxide on the current collector to Li_2_O [44]. Compared to standard coin cells, lower CEs are obtained with the operando cell. This is expected to be due to a lower cell stack pressure in the operando cell, as its geometry requires a different spring and clamping mechanism. Still, the cell provides a complementary platform for operando investigations, as the electrochemical processes happening at the interface stay the same. The second cycle was half an hour longer than the first, resulting in more plated Li ions and a notably higher CE of 62%. Measurement point 1 was recorded at open‐circuit voltage (OCV) at 2.78 V. Points 2–8 correspond to the first Li plating half‐cycle, points 9–10 to the first Li stripping half‐cycle, points 11–16 to the second Li plating half‐cycle, and points 17–20 to the second Li stripping half‐cycle.

Among all the collected spectra, Figure 4c,d show the selected spectra corresponding to the final points of each stage during two plating/stripping cycles (points 1, 8, 10, 16, and 20). From these spectra, not only can the evolution of the characteristic bands throughout the electrochemical process be observed, but also the differences arising from various incident angles. Bands around 1500–1400 cm^−1^, where spectra correspond to a 45° incident angle, exhibit a distinct band shape. In this region, CH_2_ deformation modes with different dipole orientations overlap and the relative contribution of these modes varies with local environments at different penetration depths, leading to the observed band shape changes during the electrochemical process.

The spectra from the end of the second Li plating/stripping cycle (points 16 and 20) reveal the appearance of new bands around 1610–1580 cm^−1^. The origin of these bands might be the formation of SPE degradation products upon contact with plated Li metal.

**FIGURE 5:**
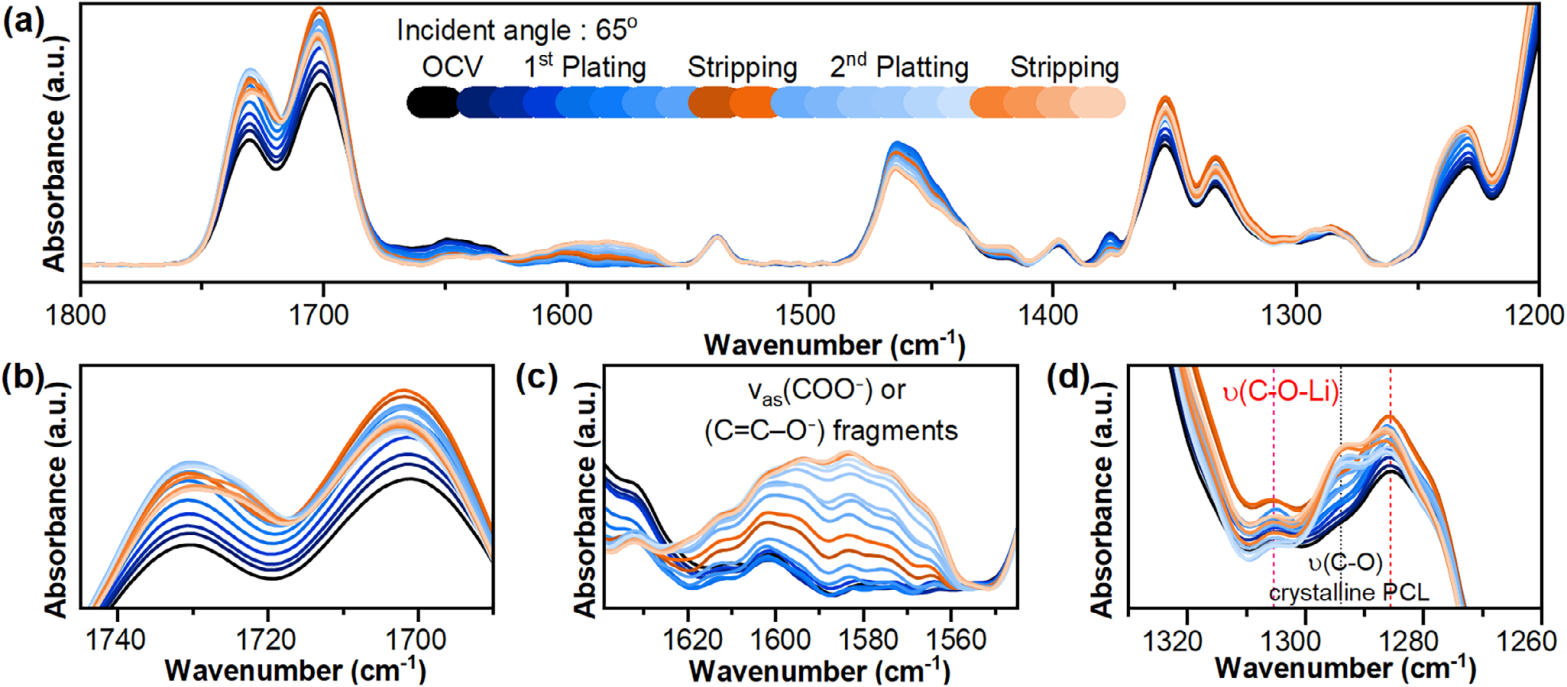
ATR‐FTIR spectra obtained at an incident angle of 65° during Li plating/stripping cycles. (a) Full spectra showing the evolution of major absorption bands throughout the electrochemical process. (b) The region of 1740–1700 cm^−1^ corresponds to ν(C═O). (c) The newly emerging band at 1610–1560 cm^−1^ is assigned to ν_as_(COO^−^) or enolate‐like (C═C─O^−^) fragments. (d) The increased absorption around 1320–1250 cm^−1^ region is attributed to ν(C─O─Li) species.

Therefore, by isolating the spectra measured at the incident angle of 65° (near the SPE|Li metal interface), shown in Figure 5, the evolution of the bands corresponding to degradation products during plating/stripping becomes more evident. In addition, the intensity evolution during the Li plating/stripping process is attributed to the electrodeposited Li metal generating a continuous mechanical stress against the SPE during Li plating, causing localized volumetric expansion of the SPE, which also improves the contact between SPE and ATR crystal. The subsequent electrochemical dissolution of lithium induces a localized volume contraction, partially relieving the interfacial stress and thus leading to a decrease in spectral intensity.

The IR bands observed around 1620–1560 cm^−1^ are primarily attributed to the formation of carboxylate species. These species originate from PCL upon contact with Li metal, triggering the cleavage of the ester bonds (C─O). The degradation products containing the RCOO^−^ functional groups correspond to the ν_as_(COO^−^) vibrational mode, which is possibly accompanied by enolate‐like (C═C─O^−^) fragments stabilized at the SPE|Li metal interface. Simultaneously, the enhancement of bands around 1320–1250 cm^−1^ region can be assigned to ν(C–O^−^)/ν(C─O─Li) vibrations of these species. Furthermore, a corresponding carbonyl species would be expected near 1720–1700 cm^−1^, however, it is likely less distinguishable due to its overlap with the main carbonyl absorption of PCL. Notably, the C═O stretching band of PCL around 1730 cm^−^^1^ broadens during the second plating/stripping cycle, which indicates a change in the chemical or physical environment of PCL. Therefore, the selected band around 1730 cm^−^^1^ is further deconvoluted, and the collected spectra were further analyzed in order to gain more detail and elucidate the interfacial mechanisms between the cl‐PCL_5_LiTFSI and the plated Li metal in the following section.

#### 2.3.1 Spectral Region Corresponding to PCL in cl‐PCL_5_LiTFSI Electrolyte

The carbonyl stretching vibration bands of PCL are located around 1730 cm^−1^ (amorphous PCL) and around 1722 cm^−1^ (crystalline PCL). These vibrational modes are particularly sensitive to structural changes, as their relative intensities vary with the crystallinity of the PCL polymer, as shown in Figure 1. During Li plating/stripping cycles, the intensity of the band around 1730 cm^−1^ was found to differ, depending on the direction of current flow and incident angle. The respective spectra are shown in Figure S8.

**FIGURE 6:**
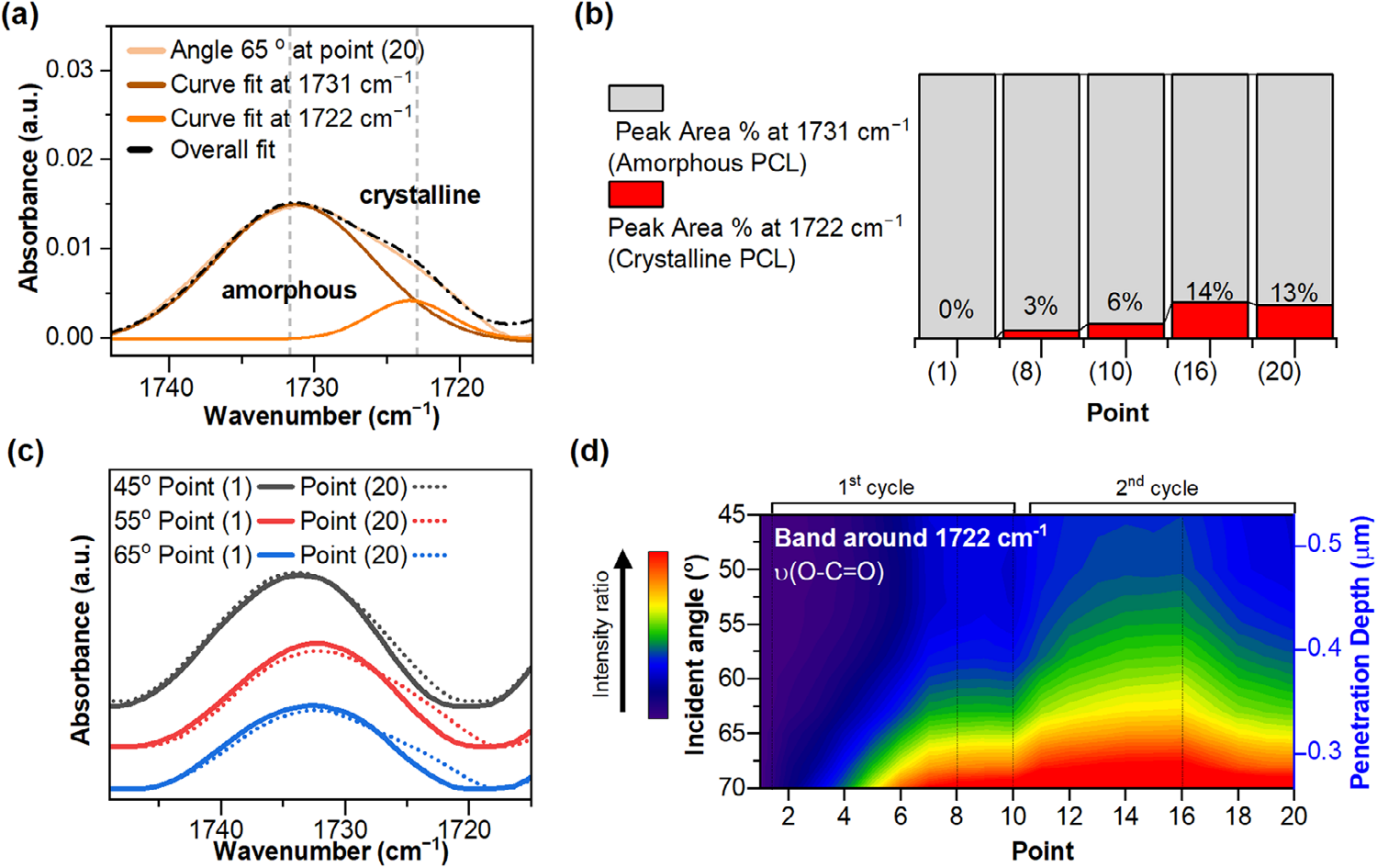
(a) Band deconvolution of the carbonyl band from the spectrum at measurement point 20 with an incident angle of 65°, showing a crystalline contribution at 1722 cm^−1^ in addition to the amorphous component at 1731 cm^−1^. (b) Relative contributions of the fitted peaks at 1731 and 1722 cm^−1^ quantified from spectra at measurement points 1, 8, 10, 16, and 20 at an incident angle of 65°. (c) Comparison of spectra at the first (point 1) and last (point 20) measurement under different incident angles, highlighting the probing depth‐dependent appearance of the crystalline shoulder at 1722 cm^−1^. (d) The intensity ratio map is made from the intensity at 1722 cm^−1^ from the spectra collected during cycling (measurement points 1−20) at incident angles of 45−70° compared to the intensity at 1722 cm^−1^ from the spectrum obtained at OCV. The map visualizes the evolution of as the band around 1722 cm^−1^ as a function of measurement point and probing depth. Red color in the map represents higher intensity of the band, and blue represents lower intensity.

An exemplary peak fitting of the spectra obtained at an incident angle of 65° at the final point of the second stripping half‐cycle (Point 20) is shown in Figure 6a. The results confirmed that the band can be deconvoluted into two peaks at 1731 and 1722 cm^−1^. Further semiquantitative analysis was carried out to evaluate the evolution of the crystalline/amorphous PCL proportion. In the analysis in Figure 6b, the spectra from points 1, 8, 10, 16, and 20 were taken, which correspond to the final states of each electrochemical step. Here, the peak area ratios clearly show an increase in crystallinity with ongoing Li plating/stripping at the interface.

To further examine whether these crystallinity changes depend on the different depth of the probed interface, spectra recorded at 45°, 55°, and 65° incident angles were compared between the OCV (point 1) and the end of the Li stripping in the second cycle (point 20). As shown in Figure 6c, the additional peak at 1722 cm^−1^ is most pronounced at higher incident angles, while only a very slight contribution can be detected at an incident angle of 45°.

To better visualize the evolution of this phenomenon, the spectral intensities at 1722 cm^−1^ were selected and converted into an intensity ratio map (Figure 6d). The ratio map can provide a more intuitive representation of the relation between the incident angles/penetration depth and the electrochemical states. The ratio heat map shows that the interfacial chain ordering accumulates and gradually extends into the nearbulk area during the Li plating/stripping process and is partially reversible during Li stripping. This result also supports the claim that the interfacial chain ordering of PCL is more dominant in the near interphase region rather than in the whole SPE. Generally, the observed evolution of a crystalline phase of PCL at the interface at 60 °C is contrary to the finding of bulk PCL being amorphous at 60 °C (Figure 1). This deviation is most likely caused by multiple factors originating from ongoing Li plating/stripping, such as arising internal pressure, changes in the electric field, and chemical reactions between SPE and Li metal. On the one hand, the phenomenon of interfacial chain ordering is induced by salt degradation products, which enhance the crystalline fraction within the electrolyte [33]. On the other hand, previous studies have shown that the coordination environment in SPEs during electrochemistry process could influence segmental dynamics [45, 46].

#### 2.3.2 Spectral Region Corresponding to LiTFSI in clPCL_5_LiTFSI Electrolyte

In this measurement, the plating/stripping voltage range was carefully kept between −0.2 to 1.5 V to minimize significant SPEs decomposition that blocks the IR beam detection. Although previous studies suggested that decomposition products of PCL may further accelerate the degradation of LiTFSI, no pronounced salt‐related degradation products or substantial decreases in band intensities were observed [36]. Nevertheless, localized or minor decomposition of the SPEs cannot be entirely excluded.

**FIGURE 7:**
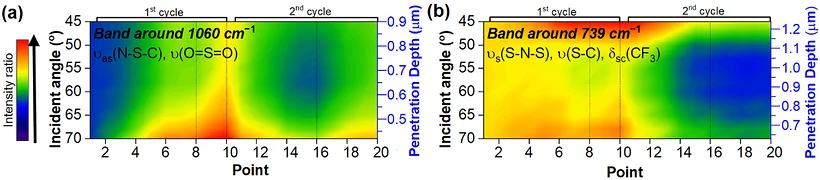
Band intensity ratio maps made from spectra collected during cycling (measurement points 1−20) at incident angles of 45–70° compared to the spectrum obtained at OCV. The maps visualize the intensity changes of selected vibrational bands as a function of measurement point and probing depth. Red color in the map represents higher intensity of the band, and blue represents lower intensity. (a) Band around 1060 cm^−1^. (b) Band around 739 cm^−1^.

Since no additional observed bands or substantial spectral changes indicate major TFSI^−^ anion decomposition, band intensity ratio maps were subsequently employed, providing more direct and informative visualization of the dynamics of TFSI^−^ anions throughout the electrochemical process. Two bands were selected for detailed analysis; one is around 739 cm^−1^ corresponding to (ν_s_(S–N–S)/ν(S–C)/δ_sc_(CF_3_)) and another around 1060 cm^−1^ corresponding to (ν_as_(N–S–C)/ν(O = S = O)), as Figure 7 shows. According to the Harrick equation, the band located at low wavenumber would correspond to a greater ATR penetration depth and thus provides information closer to the bulk SPEs, whereas the band at higher wavenumbers is probed more shallowly and therefore reflects the changes near the SPE|Li metal interface. In addition, these bands do not significantly overlap with major PCL band absorptions, and the neighboring bands around 739 cm^−1^ (i.e., 790 and 762 cm^−1^) exhibit the same trend in band intensity.

The corresponding band intensity ratio maps of these two bands are presented in Figure 7, which visualize their respective intensity changes during Li plating/stripping at different penetration depths at different incident angles.

During the first Li plating/stripping cycle (points 1‐10), a noticeable intensity increase was detected at lower penetration depths in Figure 7a, which may stem from an increasing amount of TFSI^−^ anions to compensate for the increased Li ion concentration during Li plating at the SPE|Li metal interface. Meanwhile, a distinct intensity increase can be observed at higher penetration depths (around 1.2 µm) in Figure 7b. This behavior is associated with the different mobility of anions and cations in SPEs in an electric field. Li ions migrate more slowly, as they rely on segmental motion, whereas TFSI^−^ anions migrate faster, as they interact more weakly with the polymer [47, 48]. Thus, TFSI^−^ anions can redistribute into deeper regions, leading to more pronounced intensity ratio increases at higher penetration depths. However, this phenomenon is limited to the interface‐near region instead of occurring throughout the entire SPEs, as a bulk‐scale redistribution to overcome severe concentration polarization requires excessive energy [49, 50, 51].

During the second plating process (points 11‐20), the intensity ratios of both bands decreased. This behavior could either indicate that the formed SEI is blocking the IR signal or that TFSI^−^ anions are captured within Li‐rich clusters or ordered PCL structures, reducing the population of the free TFSI^−^ anions [13, 45]. Additionally, the 1060 cm^−1^ intensity ratio map in Figure 7a indicates a different behavior in the near‐interface region. In the second Li stripping half‐cycle, a partial recovery of the intensity can be observed, indicating a relaxation of the short‐range interfacial coordination and the release of weakly confined TFSI^−^ anions [52].

**SCHEME 1:**
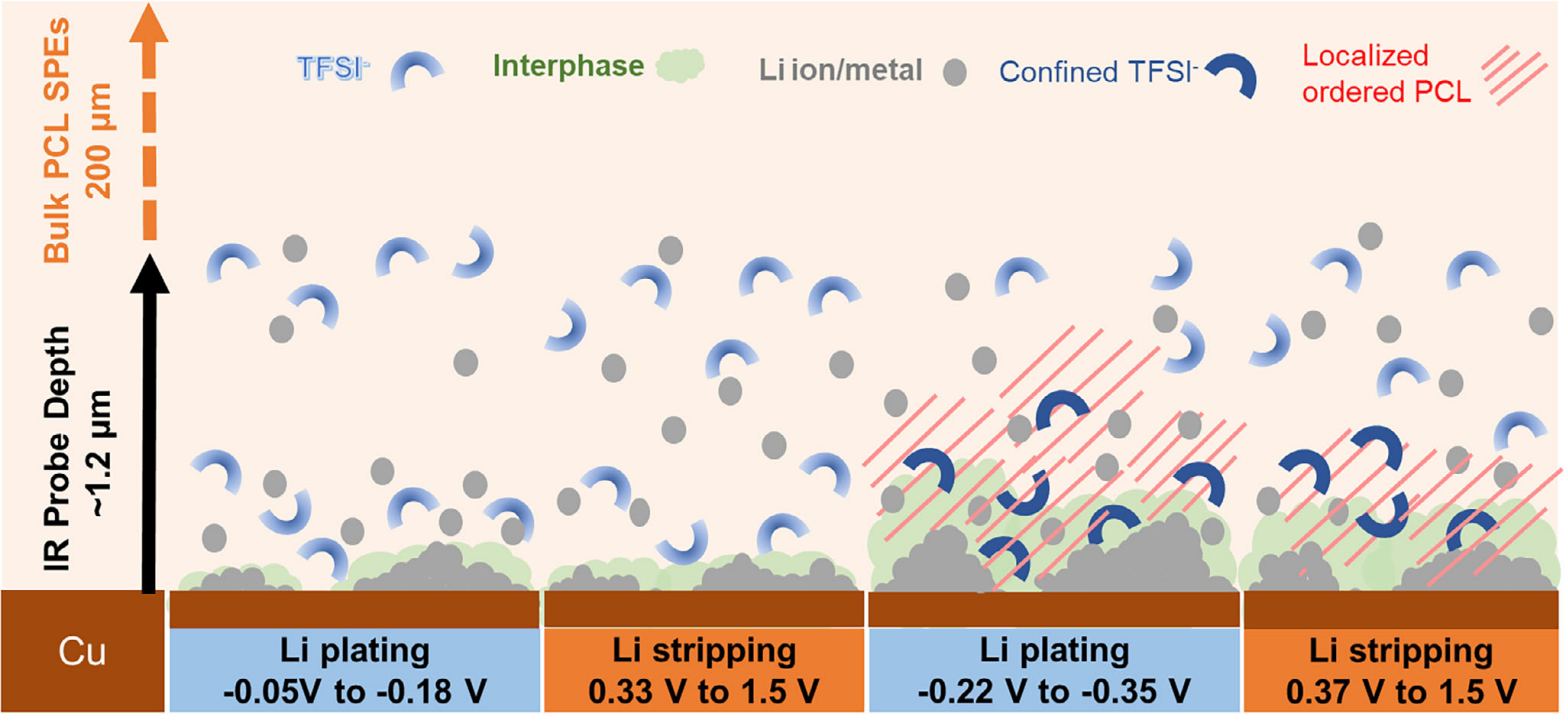
Illustration summarizing the interfacial dynamics of PCL and TFSI^−^ anions in the cl‐PCL_5_LiTFSI SPEs during Li plating/stripping cycles.

To visualize the analysis of the *operando* results and propose the interfacial evolution of PCL and TFSI^−^ anions in a more understandable way, the process is summarized in Scheme 1.

During the first Li plating/stripping, TFSI^−^ anions are observed to accumulate near the SPE|Li metal interface, consistent with charge‐compensation requirements arising from the increased local Li ion concentration. As the electrochemical process progresses, the second plating step is accompanied by the development of localized ordered PCL domains and interphase species. Moreover, TFSI^−^ anions become increasingly confined within these domains, which results in reduced intensities. Upon subsequent Li stripping, the partial relaxation of these confined structures allows weakly bound TFSI^−^ anions to be released, resulting in a partial recovery of the intensity ratio.

## 3 Conclusion

*Operando* ATR‐FTIR spectroscopy with multi‐angle penetration depth probing was used to study the structural evolution of the cl‐PCL_5_LiTFSI SPEs during Li plating/stripping. The spectra and further analysis revealed that during Li plating/stripping the carbonyl groups from PCL not only engage in dynamic coordination with Li ions but also undergo local chain ordering. While the bulk PCL matrix remains predominantly amorphous at 60 °C, this localized interfacial ordering progressively accumulates with the Li plating/stripping cycles in the interphase. The distribution of TFSI^−^ anions was found to be depth‐dependent in the spectral intensity ratio maps. Near the interphase, its coordination environment underwent dynamic rearrangements, showing relaxation during Li stripping but increasing restriction upon repeated Li plating/stripping cycles.

In addition, the electrochemical experiments demonstrate that the crosslinked PCL was found to exhibit greater stability during Li plating/stripping compared to the non‐crosslinked SPE. Nevertheless, the lifespan of PCL in the zero‐excess configuration batteries requires further improvement. Therefore, additional modification strategies such as fillers and polymer grafting are recommended to reinforce its mechanical strength and interfacial stability for further application of PCL SPEs in sustainable batteries.

## 4 Experimental

### 4.1 Membranes Preparation

Poly(ε‐caprolactone) (PCL, MW = 80,000, Sigma–Aldrich) and benzophenone (BP, 99%, Sigma–Aldrich) were pre‐dried at 40°C for 48 h under reduced pressure. Lithium bis(trifluoromethanesulfonyl)imide (LiTFSI, battery grade, E‐Lyte Innovations GmbH) was dried separately at 100°C for 48 h under reduced pressure.

To fabricate crosslinked PCL‐based SPEs (cl‐PCL_5_LiTFSI), PCL, and LiTFSI were combined at a molar ratio of 5:1, followed by the addition of 3 wt.% BP as a photo initiator. The mixture was packed in a vacuum‐sealed pouch and heated at 100°C for 12 h. The pre‐polymerized material was subsequently hot‐pressed at 100 bar and 100°C to yield membranes with a thickness of ∼0.20 mm. UV curing (UVACUBE 100, Dr. Hönle AG) was performed for 5 min to complete the crosslinking process. For the preparation of non‐crosslinked PCL‐based SPEs (PCL_5_LiTFSI), the same procedures were followed, without the addition of BP and UV curing.

### 4.2 Spectro‐Electrochemical Cell Configuration

The spectro‐electrochemical setup used in this work was adapted from a previously developed design by our group [33, 53]. To enable precise thermal regulation, the cell was modified to integrate a high‐precision heating cartridge (40 W, 24 V, E3D) capable of reaching up to 300°C, along with a PT−100 temperature probe (E3D) connected to a digital controller (ESM‐4420, EMKO). The counter electrode consisted of Li metal with a thickness of 0.50 mm. The working electrode was a copper mesh (AOT ELEC, China) with a thickness of 12 µm, pore diameter of 0.80 mm, and center‐to‐center spacing of 1.50 mm. The effective surface area of a 1 cm diameter copper mesh electrode corresponds to approximately 80% of that of a flat copper foil.

### 4.3 Coin Cell Configuration

CR2032‐type coin cells were assembled inside a dry room (dew point < 50°C, moisture level ∼0.02%). For cell assembly for ionic conductivity measurements, stainless steel components were employed as the top and bottom housings, together with two stainless steel spacers of 1.00 mm thickness and a 0.20 mm Mylar foil as a spacer in between to maintain distance between the two stainless steel spacers. For Li plating/stripping cycle measurements, the stainless‐steel spacers are 0.50 mm and 1.00 mm thick, respectively. The same copper mesh was used as the working electrode, and Li metal discs were used as the counter electrode, along with the fabricated SPEs (PCL_5_LiTFSI or clPCL_5_LiTFSI).

### 4.4 Electrochemical Procedure and Parameters

Electrochemical characterization of CR2032 coin cells was carried out using a Biologic VMP3 Potentiostat/Galvanostat/FRA system, with the cells maintained at 60°C in a constant‐temperature oven (Memmert) during testing. Prior to cycling, the cells were kept under open‐circuit conditions for 2 h to ensure thermal equilibration. Subsequently, Li plating/stripping cycles were performed at the designated current densities for 2 h per half‐cycle, with 15 min rest intervals between successive steps.

For the ionic conductivity and spectro‐electrochemical cell experiments, measurements were conducted on a PGSTAT204 potentiostat/galvanostat (Metrohm) operated with NOVA 2.1 software. The integrated heating module of the spectro‐electrochemical cell was set to 60°C to maintain a constant operating temperature. An initial OCV period of 1 h was applied for thermal equilibration, followed by Li plating/stripping at a current density of 0.05 mA cm^−^^2^.

### 4.5 Differential Scanning Calorimetry (DSC)

Differential scanning calorimetry (DSC) measurements were performed on a DSC instrument (Netzsch DSC 204 F1 Phoenix) to investigate the thermal properties and phase behavior of the pure PCL polymer and the SPEs (PCL_5_LiTFSI and cl‐PCL_5_LiTFSI). Samples weighing approximately 3–5 mg were hermetically sealed in a dry room. The measurements were carried out under a constant nitrogen flow (20 mL min^−^^1^) within a temperature range of −90°C–90°C at a heating/cooling rate of 10°C min^−^^1^.

### 4.6 ATR‐FTIR Spectroscopy

*Operando* ATR‐FTIR spectroscopy experiments were performed on an Invenio R spectrometer (Bruker) equipped with a mercury cadmium telluride (MCT) detector and a VeeMAX III automated variable‐angle reflection accessory (Pike Technologies, US), installed inside an N_2_‐purged glovebox. The electrochemical cell was mounted on a ZnSe ATR crystal (Bruker). The customized spectro‐electrochemical cell was designed using Inventor Professional 2022 software (Autodesk, US) and fabricated via 3D printing (Mars 3, Elegoo, US) with Composite‐X resin (Liqcreate, Netherlands) [53].

ATR‐FTIR spectra of reference bulk samples (Figure S1) were collected on the same spectrometer using a diamond ATR unit (Platinum ATR, Bruker) at 25°C.

For each measurement, 32 scans and 32 background scans were accumulated at a spectral resolution of 4 cm^−1^. All spectra were post‐processed with extended background subtraction (15 iterations for Figures 4c and 5 iterations for Figures 1 and 5) and H_2_O correction.

### 4.7 Penetration Depth Calculation in ATR‐FTIR Spectroscopy

The penetration depth (d_p_) of the evanescent field in ATR‐FTIR was calculated using the standard Harrick equation [42, 43]:

(1) $$d_{p}=\frac{\lambda }{\sqrt[{2\pi n_{1}}]{\sin^{2}\theta -{\left(\frac{n_{2}}{n_{1}}\right)}^{2}}}$$

d_p_: penetration depth of the evanescent field (µm)
λ: wavelength of the IR radiation in vacuum, related to the wavenumber
θ: incident angle of the IR beam relative to the surface normal of the ATR crystal.
𝑛1: refractive index of the ATR crystal (ZnSe,∼2.40).
𝑛2: refractive index of the electrolyte.

In ATR‐FTIR spectroscopy, the d_p_ of the evanescent field primarily depends on the incident angle, the vibrational wavenumber, and the refractive index contrast between the ATR crystal and the sample. In general, larger incident angles or higher wavenumbers lead to shallower penetration depths, making the spectra more sensitive to interfacial contributions. The penetration depth is defined as a characteristic decay length of the evanescent field and does not correspond to a fixed sampling thickness. Consequently, ATR spectra represent depth‐weighted averages of signals from different regions of the sample, with interfacial contributions dominating and deeper layers contributing less. The equation is derived under the assumption of a homogeneous medium with thickness much larger than d_p_, and caution should be exercised when applying it to thin‐film or highly heterogeneous systems.

## Author Contributions

**Jian‐Fen Wang**: conceptualization, investigation, validation, methodology, software, formal analysis, data curation, visualization, writing – review and editing, writing – original draft. **Matthias Weiling**: writing – review and editing, conceptualization, visualization, data curation. **Felix Pfeiffer**: conceptualization, visualization, data curation. **Marian Stan**: writing – review and editing, formal analysis, data curation, validation, conceptualization, supervision. **Martin Winter**: conceptualization, supervision, writing – review and editing. **Masoud Baghernejad**: conceptualization, funding acquisition, investigation, project administration, resources, supervision, data curation, writing – review and editing.

## Disclosure Statement

The authors acknowledge the use of Gemini Pro (accessed 2026) for initial language refinement and grammar checking to enhance the overall readability of the manuscript. All AI‐generated suggestions were critically evaluated, extensively revised, and manually adapted by the authors to ensure that the precise scientific meaning, original intent, and appropriate terminology were strictly preserved. The authors take full responsibility for the accuracy, integrity, and final content of this work.

## Conflicts of Interest

The authors declare no conflicts of interest.

## Supporting information




**Supporting File**: advs77788‐sup‐0001‐SuppMat.docx.

## Data Availability

The data that support the findings of this study are available on request from the corresponding author. The data are not publicly available due to privacy or ethical restrictions.
