# Supplementary material for: Unveiling the Interfacial Dynamics of Crosslinked Biodegradable Polycaprolactone Solid Polymer Electrolytes and Li Metal During Lithium Plating and Stripping via Operando ATR‐FTIR Spectroscopy
Source: Adv Sci (Weinh). 2026 Sep 16:e77788. Online ahead of print. doi: 10.1002/advs.77788 (PMC13583239; doi:10.1002/advs.77788)
Supplement: Supplementary file 1 [file ADVS-9999-e77788-s001.docx]

**Supporting Information**

**Unveiling the Interfacial Dynamics of Crosslinked Biodegradable Polycaprolactone Electrolytes and Li metal during Lithium Plating and Stripping *via Operando* ATR‑FTIR Spectroscopy**

Jian-Fen Wang^1^, Matthias Weiling^1^, Felix Pfeiffer^1^, Marian Stan^1^, Martin Winter^1,2^, Masoud Baghernejad^1^.*

^1^ Institute of Energy Materials and Devices, Helmholtz Institute Münster: Ionics in Energy Storage (IMD-4 / HI MS), Forschungszentrum Jülich GmbH, Corrensstraße 48, 48149 Münster, Germany

^2^ MEET Battery Research Center, Corrensstraße 46, 48149 Münster, Germany

E-mail: [b.masoud@fz-juelich.de](mailto:b.masoud@fz-juelich.de)

Figure S1. (a) FTIR spectra of PCL, LiTFSI, benzophenone, and the mixed SPEs with Li⁺:C=O ratios of 1:10 and 1:5, including their crosslinked variants (cl-PCL_10_LiTFSI, cl-PCL_5_LiTFSI). (b) Expanded spectrum (1055-970 cm⁻^1^) of PCL, LiTFSI, PCL_10_LiTFSI, and PCL_5_LiTFSI highlighting band shifts and overlapping features. (c) Expanded spectrum (860‑790 cm⁻^1^) highlighting similar spectral variations.

Table S1. IR spectra assignment of the bulk materials and the electrolytes at room temperature.

| Vibrational mode | Wavelength / cm^-1^ | | | | |
| --- | --- | --- | --- | --- | --- |
|  | PCL | LiTFSI | PCL+LiTFSI | cl-PCL electrolyte | Benzophenone |
| ν(CH_2_) | 2943 |  | 2943 | 2943 |  |
|  | 2864 |  | 2863 | 2863 |  |
| ν (O-C=O) | 1722 |  | 1722 | 1722 |  |
|  |  |  | 1700 | 1700 |  |
| ν (C=O) |  |  |  | 1656 | 1650 |
| ν (C=C) aromatic |  |  |  | 1597/1578 | 1595/1576 |
| δ(CH_2_) | 1469/1459 |  | 1469/1459 | 1469/1459 |  |
|  | 1437 |  | 1437 | 1437 |  |
|  | 1417 |  | 1417 | 1417 |  |
|  | 1395 |  | 1395 | 1395 |  |
|  | 1365 |  | 1353 | 1355 |  |
| ν_as_(O=S=O) |  | 1323 | 1332 | 1332 |  |
| ν_as_(OC-O-) | 1293 |  | 1294 | 1294 |  |
| ν_s_(C-O-C) | 1239 |  | 1239 | 1239 |  |
| ν_s_(O=S=O), ν(S-C), ν(CF_3_) |  | 1244 | 1231 | 1231 |  |
| ν(S-C),ν_as_(S-N-S) |  | 1203 | 1193 | 1193 |  |
| δ(CH_2_) | 1184 |  |  | 1184 |  |
|  | 1161 |  |  | 1161 |  |
| ν(CF_3_) |  | 1144 | 1138 | 1138 |  |
| δ(CH2) crystaline | 1093 |  | 1093 | 1093 |  |
| δ(CH2) | 1106 |  | 1105 |  |  |
| ν_as_(C-O), δ(CH_2_) | 1065 |  |  |  |  |
| ν_as_(N-S-C), ν(O=S=O) |  | 1062 | 1060 | 1060 |  |
| ν(OC-C), δ_tw_(CH_2_) | 1043 |  | 1043 | 1043 |  |
| ν(C-C) crystaline | 960 |  | 960 | 960 |  |
| δ_wag_(CH_2_) crystaline | 934 |  | 934 | 934 |  |
| ν_s_(O-C-C) crystaline | 840 |  | 840 | 840 |  |
| ν_s_(S-N-S), ν(CF_3_), ν(S-C) |  | 811 | 790 | 790 |  |
| ν(S-C), ν(CF_3_) |  | 774 | 762 | 762 |  |
| ν_s_(S-N-S), ν(S-C), δ_sc_(CF_3_) |  | 745 | 740 | 740 |  |
| δ_wag_(CH2) | 730 |  | 730 | 730 |  |
| δ_wag_(CH_2_)  δ_sc_(S-N-S), δ_sc_(CF_3_) | 710 |  | 706 | 706 | 696 |
| δ_wag_(CH_2_)  δ_sc_(S-N-S), δ_sc_(CF_3_)  δ_sc_(S-N-S), δsc(CF_3_)  δ_wag_(S-N-S), δ(O=S=O) |  |  | 653 |  |  |
|  |  | 630 | 615 | 638 | 635 |
| δ_sc_(S-N-S), δ_sc_(CF_3_)  δ_wag_(S-N-S), δ(O=S=O) |  | 607 | 600 | 600 |  |
|  | 584 |  |  |  |  |
| δ(O=S=O), δ(CF_3_) |  | 572 | 570 | 570 |  |

Table S2. Ionic conductivity of PCL_10_LiTFSI, cl‑ PCL_10_LiTFSI, PCL_5_LiTFSI, and cl‑PCL_5_LiTFSI in the temperature range of 30 ^o^C to 70 ^o^C.

| Temperature (^o^C) | **PCL_10_LiTFSI** | **cl-PCL_10_LiTFSI** | **PCL_5_LiTFSI** | **cl-PCL_5_LiTFSI** |
| --- | --- | --- | --- | --- |
|  | Ionic conductivity (µS/cm) | | | |
| 30 | 0.64 | 0.27 | 0.87 | 0.63 |
| 40 | 2.94 | 7.05 | 8.71 | 4.01 |
| 50 | 38.31 | 22.14 | 46.72 | 28.63 |
| 60 | 60.44 | 29.28 | 71.26 | 34.78 |
| 70 | 114.05 | 48.54 | 119.38 | 54.12 |

Based on the ionic conductivity measurement results of the four SPEs PCL_10_LiTFSI, cl‑PCL_10_LiTFSI, PCL_5_LiTFSI, and cl‑PCL_5_LiTFSI, it can be observed that the crosslinked SPEs exhibit lower ionic conductivity to compared to their non‑crosslinked counterparts. This decrease is primarily attributed to the crosslinked network restricting the segmental mobility. Additionally, the ionic conductivities of the SPEs with a Li:C=O ratio of 1:10 are lower than those of the SPEs with a 1:5 ratio, indicating that higher salt concentration leads to a higher density of available charge carriers.

Figure S2. DSC results of the pure PCL, PCL_5_LiTFSI and cl-PCL_5_LiTFSI SPEs measured at a heating rate of 10 °C min^-1^. Pure PCL shows a melting temperature (T_m_) of 63 ^o^C; PCL_5_LiTFSI SPE shows a T_m_ of 42 ^o^C, due to the addition of Li salts; cl‑PCL_5_LiTFSI still shows a T_m_ of 18 °C and 24 ^o^C, which may be caused by the partially remaining non-crosslinked polymer segments in SPEs

Figure S3. Temperature-dependent IR spectra measured at equilibrium temperature from 45 °C to 70 °C (color gradient from dark to light). (a) cl-PCL_5_LiTFSI and (b) PCL_5_LiTFSI SPEs.

Figure S4. Images of the Li|PCL electrolyte|Li symmetric cells after the EIS measurement. (a) The cell with non‑crosslinked electrolyte(Li|PCL₅LiTFSI|Li) shows a thick outer ring of electrolyte extruded by internal pressure. (b) The cell with non‑crosslinked electrolyte(Li|PCL₅LiTFSI|Li) shows that the electrolyte could not be separated from the Li electrodes, revealing an inhomogeneous polymer distribution, along with the presence of grayish-black lithium degradation products. (c) The cell with crosslinked electrolyte(Li|cl-PCL₅LiTFSI|Li) shows that the electrolyte retained its structural integrity, indicating superior mechanical robustness compared with the non-cross-linked counterpart.

Table S3. Fitted impedance parameters of Li|PCL_5_LiTFSI|Li and Li|cl-PCL_5_LiTFS|Li symmetric cells obtained from the shown equivalent circuit. R1 corresponds to the bulk resistance of the polymer electrolyte, while R2 + R3 represents the combined interfacial and charge-transfer resistance. EIS measurements were performed before Li deposition, immediately after plating, and during subsequent rest periods (Day 2–4).

|  | | Before | After | Day 2 | Day 3 | Day 4 |
| --- | --- | --- | --- | --- | --- | --- |
| PCL_5_LiTFSI (a) | R1 (Ω) | 131 | 117 | 99 | 75 | 57 |
|  | R2+R3 (Ω) | 156 | 202 | 302 | 337 | 359 |
| cl-PCL_5_LiTFSI  (b) | R1 (Ω) | 200 | 174 | 165 | 162 | 155 |
|  | R2+R3 (Ω) | 158 | 184 | 276 | 308 | 319 |

**Discussion of the *operando* investigation of the non-crosslinked electrolyte (PCL_5_LiTFSI)**

Figure S5. Voltage versus time profile of Li deposition/dissolution at a current density of 0.05 mA cm^−2^ in the spectro‑electrochemical cell containing Li|PCL_5_LiTFSI electrolyte|Cu substrate at 60 °C. IR spectra were recorded at each state (point 1–5).

Figure S5 presents the *operando* ATR-FTIR voltage–time profile obtained using the non‑crosslinked PCL electrolyte PCL_5_LiTFSI. Spectra at incident angles between 45° and 70° were collected (Points 1–5) during Li plating. The cell exhibited a notably low Coulombic efficiency (CE) of approximately 13%, in contrast to the first-cycle CE of ~25% observed for the crosslinked electrolyte cl-PCL_5_LiTFSI cell.

Figure S6. Baseline corrected ATR-FTIR spectra of points 1-5 representing each state of the electrochemical process at incident angle of 65°. (b) The same ATR-FTIR spectra without baseline correction.

Dur to a pronounced decrease in IR signal intensity was detected during plating (Figure S6), the measurement was intentionally limited to less than one hour. The poor electrochemical reversibility indicates chemical degradation at the plated Li metal|PCL_5_LiTFSI interface. Compared with the crosslinked cl-PCL_5_LiTFSI electrolyte, the linear PCL chains possess greater segmental mobility and expose more reactive carbonyl groups, which are prone to nucleophilic attack or reductive cleavage by metallic lithium. These reactions yield oligomeric and inorganic by-products that not only attenuate the IR signal but also consume active lithium, thereby leading to the low CE. In contrast, the crosslinked PCL network restricts chain motion and enhances mechanical integrity, effectively suppressing interfacial degradation and improving both IR stability and electrochemical reversibility.

Besides the attenuation caused by the interfacial degradation products, another major factor contributing to the pronounced decrease in peak intensity is the insufficient mechanical strength of the non-crosslinked PCL_5_LiTFSI electrolyte at 60 °C, which allows viscoelastic flow and thickness redistribution of the polymer under the applied pressure. For clarity, the main spectra were baseline-corrected (Figure S6a); however, the uncorrected raw data (Figure S6b) show a gradual elevation of the baseline over time. This baseline rise reflects enhanced optical scattering and multiple-reflection effects, indicating that the film thickness and refractive-index distribution become progressively nonuniform during the plating process, which in turn reduces the effective coupling of the evanescent field. In other words, the observed attenuation of spectral intensity arises not only from chemical degradation but also from a combination of optical-path variation and deteriorating interfacial contact.

Figure S7.ATR-FTIR spectra of points 1-5 and in different incident angle representing each state of the electrochemical process.

Figure S7 displays the *operando* ATR-FTIR spectra of the PCL_5_LiTFSI electrolyte collected at different incident angles during Li plating. Characteristic absorptions progressively attenuate from Point 1 to Point 5, demonstrating uniform signal loss throughout the polymer layer. At the early stage (Point 2), the spectra recorded at lower incident angles (45°) exhibit higher intensities because of the greater penetration depth into the still-thick polymer film. As plating proceeds, the film becomes thinner and an interfacial blocking layer forms, causing low-angle spectra to attenuate more rapidly while high-angle spectra (70°) retain higher intensity, which resulting in a reversal of the angular dependence. This inversion provides direct evidence that viscoelastic thinning and optical blocking occur simultaneously during Li deposition, both contributing to the overall IR signal decay.

Figure S8. Collected spectra during the Li plating/stripping process from low penetration depth (high incident angle of 65^o^) to high penetration depth (low incident angle of 45^o^). Blue spectra represent Li plating process, and orange spectra represent Li stripping process. The spectra at low penetration depth show an obvious band rising around 1722 cm^−1^, representing the crystalline PCL.

1.	Harrick, N. J.; Beckmann, K. H. "Internal Reflection Spectroscopy. In *Characterization of Solid Surfaces,"* Kane, P. F., Larrabee, G. B., Eds.; Springer US: Boston, MA, (1974); pp 215–245. https://doi.org/10.1007/978-1-4613-4490-2_11

2.	Smith, B. C. "*Fundamentals of Fourier Transform Infrared Spectroscopy,"* CRC press, (2011).

3.	Grdadolnik, J. "ATR-FTIR Spectroscopy: Its Advantage and Limitations," *Acta Chimica Slovenica* (2002), *49* (3), 631–642.
